# Soft tissue damage after minimally invasive THA: A comparison of 5 approaches

**DOI:** 10.3109/17453674.2010.537804

**Published:** 2010-11-26

**Authors:** Jakob van Oldenrijk, Piet VJM Hoogland, Gabriëlle JM Tuijthof, Ruby Corveleijn, Tom WH Noordenbos, Matthias U Schafroth

**Affiliations:** ^1^Department of Orthopaedic Surgery, Orthopaedic Research Center Amsterdam (ORCA), Academic Medical Centre; ^2^Department of Anatomy and Neurosciences, Vrije Universiteit Medical Centre; ^3^Department of Orthopaedic Surgery, Vrije Universiteit Medical Centre, Amsterdam, the Netherlands

## Abstract

**Background and purpose:**

Minimally invasive surgery (MIS) for hip replacement is thought to minimize soft tissue damage. We determined the damage caused by 4 different MIS approaches as compared to a conventional lateral transgluteal approach.

**Methods:**

5 surgeons each performed a total hip arthroplasty on 5 fresh frozen cadaver hips, using either a MIS anterior, MIS anterolateral, MIS 2-incision, MIS posterior, or lateral transgluteal approach. Postoperatively, the hips were dissected and muscle damage color-stained. We measured proportional muscle damage relative to the midsubstance cross-sectional surface area (MCSA) using computerized color detection. The integrity of external rotator muscles, nerves, and ligaments was assessed by direct observation.

**Results:**

None of the other MIS approaches resulted in less gluteus medius muscle damage than the lateral transgluteal approach. However, the MIS anterior approach completely preserved the gluteus medius muscle in 4 cases while partial damage occurred in 1 case. Furthermore, the superior gluteal nerve was transected in 4 cases after a MIS anterolateral approach and in 1 after the lateral transgluteal approach. The lateral femoral cutaneous nerve was transected once after both the MIS anterior approach and the MIS 2-incision approach.

**Interpretation:**

The MIS anterior approach may preserve the gluteus medius muscle during total hip arthroplasty, but with a risk of damaging the lateral femoral cutaneous nerve.

Despite the lack of convincing evidence of clinical benefits of minimally invasive surgery (MIS) approaches, as recently reported by the Canadian Agency for Drugs and Technologies in Health February 2008, an increasing number of surgeons are using MIS for THA (Coyle et al. 2008). MIS means a restricted surgical field with limited visual feedback in vivo, which makes it difficult to estimate the amount of soft tissue damage during surgery. 2 Cadaver studies have shown thatthere can be substantial damage to muscles after MIS posterior, MIS 2-incision, and MIS anterior approaches with damage extending to beyond the chosen surgical plane (Mardones et al. 2005, Meneghini et al. 2006). These studies did not cover all the MIS approaches, and there was no comparison to a conventional approach. The amount of surface area of damaged muscle was estimated using the product of the average width and the average breadth of each muscle and tendon. The disadvantage of this method is that it does not account for the pennation angle of individual muscles fibers, especially of fan-shaped muscles such as the gluteus medius.

We quantified the extent of soft tissue and nerve damage after 4 MIS approaches (MIS anterior, MIS anterolateral, MIS 2-incision, and MIS posterior) and compared it to a conventional lateral transgluteal approach. Damage to the gluteus medius was the main outcome parameter in this study, as this can cause postoperative pain at the greater trochanter and reduced abductor strength. This results in limping and a positive Trendelenburg gait, which is a frequent complication after a lateral transgluteal approach (Baker and Bitounis 1989, Pfirrmann et al. 2005, Stahelin 2006). We assumed that the clinical benefits of MIS approaches would therefore be likely to be correlated to the amount of damage to this muscle.

## Material and methods

### Cadavers, sample size, and surgeons

We selected 13 fresh frozen human adult cadavers (7 female) without previous hip surgery and without known bone deformities or muscular disorders. We excluded cadavers with skeletal abnormalities on preoperative anteroposterior pelvic radiography. The cadavers were transected at the level of the iliac crest/L4 and distally at the proximal one-third of the lower leg. Before surgery, the cadavers were thawed at room temperature and weighed. From 25 hips, 5 hips were assigned to 1 group, to reach similar weight distributions, followed by random allocation of each group to 1 approach. There were 2 males in the lateral transgluteal, MIS 2-incision, and MIS posterior groups, 3 males in the MIS anterior group, and 4 males in the MIS anterolateral group. The calculation of sample size was based on the assumption that a lateral transgluteal approach results in damage to approximately one-third of the gluteus medius. Previous studies found a mean damage of 15% after a MIS 2-incision approach and even less damage after the MIS anterior and MIS posterior approaches (Mardones et al. 2005, Meneghini et al. 2006). This corresponds to a reduction of damage of more than 50% using MIS approaches. A sample size of 5 in each group would have 80% power to detect a difference in means of 18% (the difference between the MIS 2-incision mean of 15% and the lateral approach mean of 33%), assuming that the common standard deviation to be 8.5.

5 surgeons with experience in 1 specific approach each performed 5 operations using that particular approach; the surgeons had each performed between 200 and 600 THAs using their specific approach. We studied the MIS anterior approach (Rachbauer 2006), the MIS anterolateral approach (Bertin and Rottinger 2004), the MIS 2-incision approach (Berger and Duwelius 2004), the MIS posterior approach (Sculco and Boettner 2006), and the lateral transgluteal approach (Bauer et al. 1979). Similarly to an actual surgery setting, the surgeons were assisted by 2 assistants and they were provided with instruments and implants to which they were accustomed.

### The lateral transgluteal approach

This approach was done with the cadaver in the lateral decubitus position (Bauer et al. 1979). The skin incision, measuring 10–15 cm, was centered over the greater trochanter and was followed by incision of the iliotibial tract. A longitudinal incision was made over the midpart of the tendon of the gluteus medius and vastus lateralis. The ventral part of the gluteus medius tendon was detached and moved ventrally, while attempting to preserve the connection between the ventral part of the tendon of the gluteus medius muscle and the vastus lateralis muscle. The incision was extended proximally parallel to the fibers of gluteus medius. The anterior capsule was excised. After osteotomy of the femoral neck, the femoral head was dislocated and extracted. The acetabulum was under-reamed by 1 or 2 mm, depending on fit, and a cementless press-fit cup was inserted (Trilogy; Zimmer, Warsaw, IN). The medullar canal was visualized by adduction and external rotation of the leg. The dorsal part of the gluteus medius tendon was protected by a Hohmann retractor. After a box osteotomy followed by a series of rasps, the final cementless straight stem was inserted (Alloclassic; Zimmer, Warsaw, IN).

### The MIS anterior approach

This was performed with the cadaver in supine position (Rachbauer, 2006). The skin incision (< 10 cm) originated approximately 2 finger-breadths lateral and distal to the anterior superior iliac spine and extended distally in the direction of the patella. After dissecting the subcutaneous tissue, the surgical interval was bluntly dissected along the medial border of the tensor fasciae latae (TFL) muscle to reduce the risk of damage to the lateral femoral cutaneous nerve. The anterior capsule was excised followed by a double osteotomy of the femoral neck, removal of the femoral neck fragment, and finally the femoral head. The acetabulum was under-reamed by 1–2 mm and a cementless press-fit cup was inserted (seleXys; Mathys, Bettlach, Switzerland). For femoral preparation, the leg was put in external rotation, hyperextension, and adduction, with flexion of the knee resulting in a figure-of-4 position. To allow adequate femoral exposure, a dorsolateral soft tissue release was performed. Curved rasps were used for broaching of the canal, followed by insertion of the press-fit anatomically-shaped stem (TwinSys; Mathys, Bettlach, Switzerland).

### The MIS anterolateral approach

This was performed with the body in a lateral decubitus position (Bertin and Rottinger, 2004). The intermuscular plane between the TFL and the gluteus medius muscle was palpated, followed by a skin incision (< 10 cm) running from the anterior tubercle of the greater trochanter towards the anterior superior iliac spine. The hip capsule was reached by blunt dissection between the TFL and gluteus medius muscles. The anterior capsule was excised and a double femoral neck osteotomy was performed with the leg in external rotation. The first cut was placed directly subcapital, while the final cut was placed more distal on the intertrochanteric line. The resulting wedge-shaped disc was removed, thereby creating enough space to remove the femoral head. The head was dislocated and extracted. The acetabulum was under-reamed by 1–2 mm, followed by insertion of the press-fit cementless cup (Trilogy; Zimmer). Femoral exposure was achieved with the hip in hyperextension, adduction, and external rotation. A box osteotomy was performed, followed by a series of rasps and the introduction of the final cementless straight stem (Alloclassic; Zimmer).

### The MIS 2-incision approach

This was performed with the cadaver in supine position (Berger and Duwelius, 2004). Fluoroscopy was used to determine the position of the skin incision. The anterior incision was centered on the midline of the femoral neck, originating at the base of the head, extending to the intertrochanteric line (< 5 cm). Further dissection was through the interval between the TFL and the sartorius muscle along the medial border of the TFL. After a double femoral neck osteotomy, the femoral head was dislocated and extracted. The acetabulum was under-reamed by 1–2 mm. Aided by fluoroscopy, the cup was inserted (Trilogy; Zimmer). For femoral exposure, the leg was adducted and under fluoroscopic guidance a posterior incision in the lateral buttocks was made, parallel to the long axis of the medullar canal (< 5 cm). By digital blunt dissection, the interval between the gluteus medius and piriformis muscle was found, enabling a longitudinal incision of the posterolateral capsule from inside out through which the medullar canal could be reached. After reaming the canal, broaching was performed under fluoroscopic guidance, followed by insertion of the final straight stem (VerSys Fiber Metal Taper; Zimmer).

### The MIS posterior approach

This approach was performed with the cadaver in the lateral decubitus position (Sculco and Boettner, 2006). The skin incision was made along the posterior border of the greater trochanter (< 10 cm). After incision of the gluteus maximus, the anterior portion was retracted anteriorly, thereby revealing the underlying external rotators and the piriformis muscle. The external rotator conjoined tendon (consisting of the inferior and superior gemelli muscles and the internal obturator muscle) and the piriformis muscle were released and reflected posteriorly, followed by posterior capsulotomy. Subcutaneous Steinmann pins and a Charnley self-retaining retractor were used for full exposure. The head was dislocated followed by osteotomy of the femoral neck. The acetabulum was under-reamed by 1–2 mm, after which the press-fit cementless cup was inserted (Trilogy; Zimmer). Preparation of the femur was achieved by positioning the leg in maximal internal rotation and adduction followed by opening of the canal with a box chisel and sequential reaming of the shaft. Finally, a cementless straight stem was inserted (Alloclassic; Zimmer).

Released structures or damaged structures intended for re-attachment were marked with a blue Vicryl 2.0 suture.

### Data acquisition

We applied several precautions to minimize the risk of postoperative damage. First, all cadavers were immediately fixed postoperatively by submersion in a 10% formalin solution. This made the tissue less vulnerable to decay and handling, and enabled us to slowly and carefully dissect the soft tissue. Furthermore, all dissections were performed according to a dissection protocol initiating dissection away from any potentially valuable tissue and working towards the surgical plane (see Supplementary data). Dissections were performed by a clinical anatomist (PVJMH) assisted by 2 independent assessors (JVO, TWHN) who were not involved in the surgical process.

**Figure 1.**
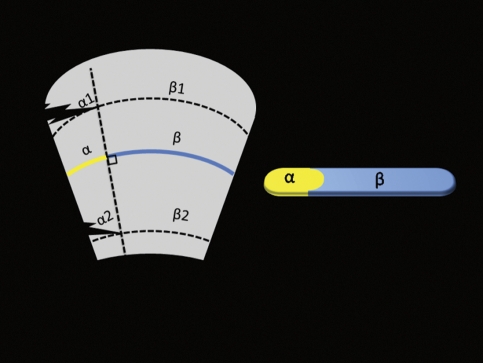
Schematic representation of the muscle damage measurements relative to the midsubstance cross-sectional surface area (MCSA). A relatively large damaged area at the origo (α1) may yield the same percentage of damaged MCSA (α) as a relatively small area at the musculotendinous insertion (α2).

**Figure 2.**
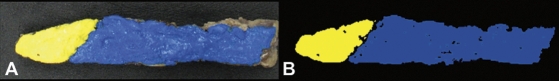
Color segmentation of a cross-sectional slice of a damaged gluteus medius muscle. Panel A is an image of the color-stained slice. Panel B shows the same slice after color segmentation.

To enable accurate objective comparison between the surgical approaches, measurement of muscle damage was normalized as follows. The assumption was made that only disruption of muscle fibers diminishes muscle function, as opposed to dissection between muscle fibers. Muscle damage was thereby defined as the percentage of macroscopic disruption of muscle fibers compared to the total amount of muscle fibers. As the pennation angle and shape of the muscle is crucial in assessing the direction of the muscle fibers, and as damage occurred on various locations, measurement of muscle damage was normalized by assessing the percentage of damage relative to the midsubstance cross-sectional surface area (MCSA). The MCSA was defined as the cross-sectional area bisecting the muscle fibers halfway between origo and insertion. In this way, the MCSA is always perpendicular to the direction of the muscle fibers. This implies that a relatively large damaged area at the origo (α1) may yield the same percentage of damaged MCSA (α) as a relatively small area at the musculotendinous insertion (α2) (Figure 1). Thus, relatively speaking, both α1 and α2 result in the same percentage of disrupted muscle fibers at the level of the MCSA α. During the dissection, a cross-sectional slice was made perpendicular to the direction of the muscle fibers at the MCSA level for each muscle with macroscopic damage. The damaged area was colored yellow and the undamaged area was colored blue with latex paint (Figure 2A). The resulting slices were photographed with an 8-megapixel camera (Canon Inc., Ohta-ku, Tokyo, Japan) (Figure 2A). The ratio of blue and yellow pixels was detected semi-automatically with customized software programmed in MATLAB Version 7.0.4.365 (R14) (The Mathworks Inc, Natick, MA) (Figure 2B). The blue and yellow color range along the RGB color axis was defined by selecting a sample of 10 blue regions (10 × 10 pixels), and 10 yellow regions (10 × 10 pixels) in each photograph. After defining a bounding box for segmentation, all pixels within the bounding box with either an average yellow or an average blue color and a range of 1.7 times the standard deviation along the RGB color axis were automatically counted (Gonzales and Woods, 2002). The percentage of yellow pixels in proportion to the total number of colored pixels (the total sum of yellow and blue pixels) represented the damaged muscle area at the MCSA level. The inter- and intraobserver reliability was reflected by an intraclass coefficient of greater than 0.9 for each muscleThe primary outcome measure was the amount of damaged MCSA of the gluteus medius muscle. Secondarily, the amount of MCSA damage was assessed for the gluteus maximus, gluteus minimus, TFL, quadratus femoris, rectus femoris, and sartorius muscle. Damage to the external rotators (the piriformis muscle, internal obturator muscle, and inferior and superior gemelli muscles) was assessed dichotomously since they were either undamaged or completely released. The iliofemoral and ischiofemoral ligaments were categorized as being completely released, partially damaged, or intact.

### Statistics

Due to small sample sizes and skewed distributions, data were described by medians and accompanying ranges and tested non-parametrically. Differences in MCSA damage between MIS approaches and the lateral transgluteal approach were determined using a Kruskal-Wallis test. For the comparison of the primary variable—gluteus medius muscle damage—a p-value of ≤ 0.05 was considered to be statistically significant. For testing of the difference between the lateral transgluteal approach and the MIS approaches (with secondary variables gluteus maximus, gluteus minimus, TFL, quadratus femoris and rectus femoris muscles), the p-value was adjusted for multiple comparisons to a p-value of ≤ 0.01 by dividing 0.05 by 5 (Bonferroni correction). Damage to the remaining structures is presented as frequencies without statistical comparison with the conventional approach.

## Results

### Gluteus medius muscle damage (Tables 1 and 2)

The median gluteus medius MCSA muscle damage after a lateral transgluteal approach was 22% (6–40). The gluteus medius muscle was completely preserved in 4 of the 5 cases using a MIS anterior approach. The difference, however, between it and the lateral transgluteal approach was not statistically significant due to 1 outlier with a damaged MCSA of 35%. The median damage to the gluteus medius MCSA was 18% (6–27) after a MIS anterolateral approach, 26% (14–40) after a MIS 2-incision approach, and 18% (0–22) after a MIS posterior approach. None of these approaches showed statistically significantly less damage to the MCSA of the gluteus medius muscle than with the lateral transgluteal approach

**Table 1.** Percentage of gluteus medius muscle damage in 5 hips during each approach

Hip	Lateral transgluteal	MIS anterior	MIS anterolateral	MIS 2-incision	MIS posterior
1	6	35	27	29	22
2	22	0	14	24	0
3	16	0	6	40	18
4	32	0	23	26	0
5	40	0	18	14	22
Median	22	0	18	26	18

**Table 2.** Comparison of the amount (%) of MCSA muscle damage between the lateral transgluteal approach and the 4 MIS approaches. Values are median (range)

Muscle	Lateral transgluteal	MIS anterior	MIS anterolateral	MIS 2-incision	MIS posterior	p-value (K-W test)
M. gluteus medius	22 (6–40)	0 (0–35)	18 (6–27)	26 (14–40)	18 (0–22)	0.1
M. gluteus maximus	1 (0–3)	0 (0–0)	0 (0–0)	0 (0–5)	2 (0–5)	0.1
M. gluteus minimus	48 (0–100)	31 (0–54)	27 (0–46)	12 (0–21)	24 (0–45)	0.5
M. quadratus	0 (0–0)	0 (0–20)	0 (0–0)	0 (0–0)	70 (13–100)	< 0.01
M. rectus femoris	0 (0–0)	0 (0–24)	0 (0–0)	0 (0–0)	0 (0–0)	0.8
M. tensor fascia latae	0 (0–26)	35 (16–100)	0 (0–0)	44 (7–49)	0 (0–0)	0.01

### Additional muscle damage

None of the MIS approaches showed statistically significantly less damage to the MCSA of the gluteus maximus, gluteus minimus, quadratus, rectus femoris, and TFL muscles than the lateral transgluteal approach. Instead, there was more damage to the quadratus and the TFL muscles after the MIS approaches (p ≤ 0.01). The median quadratus MCSA muscle damage after a MIS posterior approach was 70% (13–100). The median TFL MCSA muscle damage was 35% (16–100) after a MIS anterior approach and 44% (7–49) after a MIS 2-incision approach. The external rotator muscles were transected in 1 hip each after the lateral transgluteal approach and the MIS anterior approach, and in all hips after the MIS posterior approach. The MIS 2-incision approach resulted in a transection of the gemellus inferior in 2 hips, while the remaining external rotator muscles were preserved. The MIS anterolateral approach was the only approach with complete preservation of all external rotator muscles (Table 3).

**Table 3.** The frequency of released external rotators and transected nerves for each of the 5 approaches

	Lateral transgluteal	MIS anterior	MIS anterolateral	MIS 2-incision	MIS posterior
	(n)	(n)	(n)	(n)	(n)
*Muscle release*					
M. piriformis	1	0	0	0	3
M. gemellus superior	1	1	0	0	5
M. gemellus inferior	1	1	0	2	5
M. obturator internus	1	1	0	0	5
*Transected nerves*					
Gluteus superior nerve	1	0	3	0	0
Lateral femoral cutaneous nerve	0	1	0	1	0
Sciatic nerve	0	0	0	0	0

### Nerve damage (Table 3)

**Figure 3.**
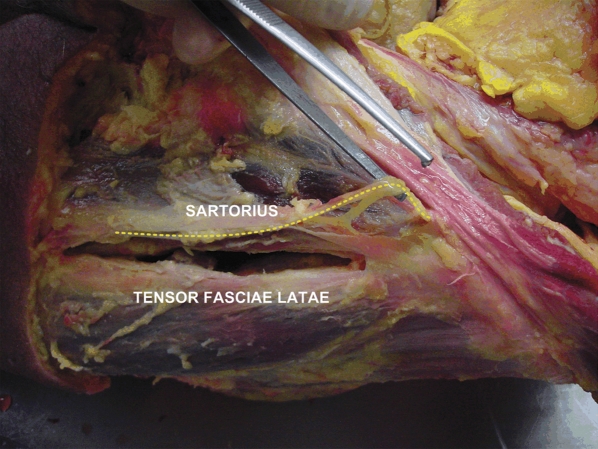
The course of the lateral femoral cutaneous nerve (yellow dotted line) is shown in relation to the interval between the sartorius muscle and TFL muscle in the MIS anterior and the MIS 2-incision approaches.

The superior gluteal nerve was transected in 1 hip after a lateral transgluteal approach, in the space between the gluteus minimus and medius muscles. After the MIS anterolateral approach, there were 3 cases with complete transection of the superior gluteal nerve in the surgical interval between the gluteus medius and the TFL muscles. The lateral femoral cutaneous nerve was fully transected along the lateral border of the sartorius muscle in 1 case each after a MIS anterior approach and a MIS 2-incision approach (Figure 3). None of the cases had any damage to the sciatic nerve.

### Ligament damage

The iliofemoral ligament was partially or completely damaged in all hips. The ischiofemoral ligament was partially or completely damaged in all hips after the lateral transgluteal and MIS posterior approaches, in 4 hips after the MIS anterior and MIS 2-incision approaches, and in 1 hip after the MIS anterolateral approach.

## Discussion

Our study has several limitations. Using fresh frozen cadavers is always an approximation of in vivo surgery. Freezing of fresh tissue without cryoprotection leads to considerable damage to the tissue at the microscopic level. Thus, the mechanical qualities of the tissue might have changed, possibly resulting in exaggeration of the muscle damage seen. However, since this was a comparative study, it would affect all approaches equally. The body weight of the cadavers varied, thus, some may have thawed out faster than others, resulting in differences in the texture of soft tissue. Despite there being a similar median specimen weight of 20–23 kg, the distribution was relatively smaller in the MIS anterolateral (21–35 kg), MIS 2-incision (18–32 kg), and MIS anterior (21–34 kg) groups as compared to the lateral (11–40 kg) and MIS posterior (11–40 kg) groups. In addition, sex distribution varied somewhat between groups. The use of fresh frozen cadavers limits assessment of neurological damage microscopically. Thus, we only determined complete transection of nerves. Furthermore, we did not measure the location of nerves relative to the surgical plane, nor to any anatomical landmarks; we were therefore not able to describe anatomical variations. Surgical expertise may have played a major role in the amount of surgical damage. Even though all participating surgeons had ample experience with one specific approach, the resulting amount of damage may have varied between surgeons, independently of the type of approach.

The gluteus medius muscle was the main structure of interest. Damage to this muscle could cause postoperative pain at the greater trochanter and reduced abductor strength, resulting in limping and a positive Trendelenburg gait (Baker and Bitounis 1989, Pfirrmann et al. 2005, Stahelin 2006). MRI one year after a lateral transgluteal approach showed that damage to the gluteus medius muscle and tendon was common in symptomatic patients (Pfirrmann et al. 2005). A study assessing the postoperative integrity of the gluteus medius tendon aponeurosis on the greater trochanter after THA found that Trendelenburg gait increased only in patients with a separation of more than 2.5 cm (Svensson et al. 1990). This suggests that moderate damage to the gluteus medius muscle or tendon might be readily compensated for.

The iliofemoral ligament provides passive stabilization of the hip in extension, and restricts hyperextension. The ischiofemoral ligament provides restriction of internal rotation and restricts adduction in flexion (Hewitt et al. 2002). Release of the ischiofemoral ligament, together with external rotators, may increase the risk of dislocation (Suh et al. 2004). In our study, the MIS posterior approach required intentional release of the ischiofemoral ligament and external rotators, which were marked for repair. In contrast, damage to these structures after the MIS anterior, lateral transgluteal, and MIS 2-incision approaches was unintentional and not marked for repair. Particularly when using an anterior approach, exposure of the femoral medullar canal is often difficult and it often requires a posterolateral release, with subsequent risk of releasing the external rotators and the ischiofemoral ligament (Meneghini et al. 2006, Rachbauer 2006). Conversely, the MIS anterolateral approach did not result in a release of any posterior structures.

The superior gluteal nerve innervates the gluteus minimus and medius and tensor fasciae latae muscles. It is still unclear whether postoperative Trendelenburg gait is caused by direct damage to the abductor muscles or by damage to the superior gluteal nerve (Ramesh et al. 1996, Kenny et al. 1999, Picado et al. 2007). Several cadaver studies determined a possible safe zone when using a lateral approach for the superior gluteal nerve, between 3 and 5 cm above the greater trochanter (Jacobs and Buxton 1989, Lavigne and Loriot de Rouvray 1994). Perioperative electromyographic (EMG) studies have, however, indicated that compression and traction forces may cause damage beyond this safe zone (Siebenrock et al. 2000).

EMG and cadaver studies have shown that the tensor fasciae latae muscle is an important flexor during the swing phase and an important abductor during the full stance phase of gait (Gottschalk et al. 1989). It balances the weight of the trunk and the non-weight bearing leg during walking. In our study, the branch of the superior gluteal nerve leading to the tensor fasciae latae muscle was transected in 4 cases after the MIS anterolateral approach, which could lead to denervation of this muscle. However, a recent cadaver study by Ince et al. (2007) found a distal inferior branch of the gluteus superior nerve towards the tensor in half of the cases, indicating that damage to the superior branch of the superior gluteal nerve may only lead to partial denervation of the tensor in these cases.

The lateral femoral cutaneous nerve was transected once after both the MIS anterior approach and the MIS 2-incision approach. Since the nerve lies in close proximity to the lateral border of the sartorius muscle, the use of blunt dissection along the medial border of the tensor may reduce the risk of transecting this nerve. However, traction or compression forces on the edges of the wound by retractors may be 3 times higher when using MIS approaches than with conventional approaches, thereby potentially increasing the risk of nerve damage by compression (Noble et al. 2007).

Whether or not MIS total hip arthroplasty really leads to accelerated rehabilitation and less postoperative pain than conventional total hip arthroplasty is still under debate (Coyle et al. 2008, Sharma et al. 2009). Furthermore, it is not known whether MIS total hip arthroplasty gives less soft tissue damage than conventional total hip arthroplasty (Mardones et al. 2005, Meneghini et al. 2006). The purpose of our study was to quantify the amount of muscle damage, particularly to the gluteus medius muscle, through 4 MIS approaches, and to compare this to the damage after a lateral transgluteal approach. We did not find any statistically significant difference in the amount of gluteus medius muscle damage between the lateral transgluteal and MIS approaches, although the MIS anterior approach completely preserved the gluteus medius muscle in 4 of 5 cases. This could be due to the small sample sizes in our study, with the risk of a type-II error.

We conclude that the MIS anterior approach could possibly preserve the gluteus medius muscle during total hip arthroplasty, but with the risk of damaging the lateral femoral cutaneous nerve.

## Supplementary data

For dissection protocol, see www.actaorthop.org, identification number 3730.
