# Supplementary material for: Soft tissue damage after minimally invasive THA: A comparison of 5 approaches
Source: Acta Orthop. 2010 Nov 26;81(6):696–702. doi: 10.3109/17453674.2010.537804 (PMC3216079; doi:10.3109/17453674.2010.537804)
Supplement: Supplementary file 1 [file ORT-1745-3674-81-696-s3730.pdf]

Supplementary article data

Soft tissue damage after minimally invasive THA

A comparison of 5 approaches

Jakob van Oldenrijk[1], Piet V J M Hoogland[2], Gabriëlle J M Tuijthof[1], Ruby Corveleijn[3], Tom W H Noordenbos[2], and Matthias U Schafroth[1]

[1]Department of Orthopaedic Surgery, Orthopaedic Research Center Amsterdam (ORCA), Academic Medical Centre; [2]Department of Anatomy and Neurosciences, Vrije Universiteit Medical Centre; [3]Department of Orthopaedic Surgery, Vrije Universiteit Medical Centre, Amsterdam, the Netherlands
Correspondence: jakobvanoldenrijk@gmail.com
Submitted 09-10-07. Accepted 10-04-10

Appendix 1 – Dissection protocol

The dissection was initiated away from any potentially liable tissue followed by slowly working towards the surgical plane. As soon as any surgical damage was visualized this was immediately marked with yellow latex, which was fixated with an acidic fluid. Any damage occurring after the coloring and staining process was considered nonsurgical and not included in the analysis. The dissection involved a posterior incision through the gluteus maximus followed by blunt dissection through the sulcus glutealis between the gluteus maximus and medius muscles. By deflecting the gluteus maxmimus, the sciatic nerve and posterior muscles around the hip joint were exposed. The gluteus medius was released from the iliac crest, followed by blunt dissection between the gluteus medius and minimus muscles and exploration of the posterior portion of the superior gluteal nerve. This revealed the posterior insertion of the gluteus medius and minimus tendon. After observing the integrity of the external rotator tendon insertions, they were released from the greater trochanter, thereby revealing the posterior capsule. The posterior incision was extended distally and medially towards the medial aspect of the patella, followed by removal of the skin, thereby exposing the anterior muscles. The anterior location of the lateral femoral cutaneous nerve, near the anterior superior iliac spine, is explored and followed distally between the sartorius and tensor fascia latae. The sartorius muscle is released from its attachment to the medial surface of the tuberositas tibiae and its body is inspected. The tensor fascia latae is incised distally from the wound at the iliotibial tract and deflected laterally, thereby exposing the full body of the tensor fascia latae as well as the anterior insertion of the gluteus medius and minimus on the greater trochanter, By removing these muscles in one layer the anterior portion of the superior gluteal nerve and its course towards the tensor fascia latae, as well as the anterior hip capsule may be inspected.

Open Access - This article is distributed under the terms of the Creative Commons Attribution Noncommercial License which permits any noncommercial use, distribution, and reproduction in any medium, provided the source is credited.
DOI 10.3109/17453674.2010.537804
